# Developmental Exposure to Polychlorinated Biphenyls Influences Stroke Outcome in Adult Rats

**DOI:** 10.1289/ehp.10828

**Published:** 2008-01-14

**Authors:** Suzan Dziennis, Dongren Yang, Jian Cheng, Kim A. Anderson, Nabil J. Alkayed, Patricia D. Hurn, Pamela J. Lein

**Affiliations:** 1 Department of Anesthesiology and Peri-Operative Medicine and; 2 Center for Research on Occupational and Environmental Toxicology, Oregon Health & Science University, Portland, Oregon, USA; 3 Environmental & Molecular Toxicology, Oregon State University, Corvallis, Oregon, USA

**Keywords:** Aroclor 1254, cerebral ischemia, developmental origins of adult disease, polychlorinated biphenyls, stroke

## Abstract

**Background:**

The “developmental origins of adult disease” hypothesis was originally derived from evidence linking low birth weight to cardiovascular diseases including stroke. Subsequently, it has been expanded to include developmental exposures to environmental contaminants as risk factors for adult onset disease.

**Objective:**

Our goal in this study was to test the hypothesis that developmental exposure to poly-chlorinated biphenyls (PCBs) alters stroke outcome in adults.

**Methods:**

We exposed rats to the PCB mixture Aroclor 1254 (A1254) at 0.1 or 1 mg/kg/day in the maternal diet throughout gestation and lactation. Focal cerebral ischemia was induced at 6–8 weeks of age via middle cerebral artery occlusion, and infarct size was measured in the cerebral cortex and striatum at 22 hr of reperfusion. PCB congeners were quantified in brain tissue by gas chromatography with microelectron capture detection, and cortical and striatal expression of *Bcl2* and *Cyp2C11* were quantified by quantitative reverse transcriptase-polymerase chain reaction.

**Results:**

Developmental exposure to A1254 significantly decreased striatal infarct in females and males at 0.1 and 1 mg/kg/day, respectively. Predominantly *ortho*-substituted PCB congeners were detected above background levels in brains of adult females and males exposed to A1254 at 1 but not 0.1 mg/kg/day. Effects of developmental A1254 exposure on *Bcl2* and *Cyp2C11* expression did not correlate with effects on infarct volume.

**Conclusion:**

Our data provide proof of principle that developmental exposures to environmental contaminants influence the response of the adult brain to ischemic injury and thus represent potentially important determinants of stroke susceptibility.

Neonatal factors can cause latent functional changes that increase susceptibility to disease and/or dysfunction later in life. This hypothesis of the “developmental origins of adult disease” was originally derived from studies that identified stroke as one of several cardiovascular diseases associated with low birth weight (McMillen and Robinson 2005; Morley 2006). Subsequently, brief separations of infant mice from their mothers were demonstrated to increase their sensitivity to cerebral ischemic injury as adults, an effect mediated by functional changes in the hypothalamic–pituitary axis and inflammatory response (Craft et al. 2006). Recent evidence linking developmental exposure to environmental contaminants of concern to neurodegenerative diseases (Barlow et al. 2007; Bolin et al. 2006; Zawia and Basha 2005) suggests a third category of neonatal factors that influences susceptibility of the adult brain to injury, but whether these factors influence adult sensitivity to stroke has not been investigated.

Polychlorinated biphenyls (PCBs) are organic compounds that were used mostly as coolants and lubricants from the 1930s until 1977, when they were banned. Despite the ban, PCBs persist in environmental samples, and high residue levels are still detected in foods, particularly fish (Kostyniak et al. 2005; Stewart et al. 1999), and in human tissue samples (Chu et al. 2003; Covaci et al. 2002; DeCaprio et al. 2005; Jursa et al. 2006). Epidemiologic and laboratory evidence suggests that PCBs increase the incidence of risk factors for stroke such as diabetes (Everett et al. 2007; Longnecker and Daniels 2001; Vasiliu et al. 2006) and vascular disease (Carpenter 2006; Hennig et al. 2005); also, living near hazardous waste sites contaminated with PCBs was recently linked to increased stroke incidence (Shcherbatykh et al. 2005). However, these studies did not address whether PCBs influence stroke outcome (the extent of damage occurring after a stroke) nor did they consider the contribution of developmental exposure to PCBs in determining adult susceptibility to cerebral ischemia. The developing nervous system is particularly sensitive to PCBs (Carpenter 2006; Schantz et al. 2003), and in experimental models, exposure of the developing brain to PCBs alters gene expression (Basha et al. 2006) and seizure susceptibility (Pessah IN, personal communication) in the adult brain.

Factors known to influence stroke outcome include estrogen (Hurn et al. 2005) and preconditioning, a well-described phenomenon in which exposure to a mild ischemic or noxious stress up-regulates endogenous anti-oxidant and anti-inflammatory mechanisms to protect the brain against subsequent more severe ischemic injury (Koerner and Alkayed 2007). Although mechanisms of preconditioning are not completely understood, *de novo* gene expression in the brain is clearly required. *Bcl2* (B-cell leukemia/lymphoma 2) and *Cyp2C11* (cytochrome P450 2C11) are two genes whose up-regulation has been functionally implicated in preconditioning (Koerner and Alkayed 2007). Bcl2 protects neurons against ischemic injury via its anti-apoptotic activity (Kitagawa 2007; Mattson 2007), and CYP2C11 metabolizes arachidonic acid to expoxyeicosatrienoic acids, which are thought to protect against cerebral ischemia via vasodilatory, anti-inflammatory and antioxidant activities (Koerner and Alkayed 2007). Developmental PCB exposure modulates systemic (Meerts et al. 2004) and brain (Colciago et al. 2006) levels of estrogen, increases *Bcl2* expression in adult testes (Hsu et al. 2007), and increases expression and activity of hepatic *Cyp2C11* (Chen et al. 1992; Kramer et al. 1999). These observations suggest that developmental exposure to PCBs may alter stroke outcome. We tested this hypothesis using a well-established animal model of focal cerebral ischemia to quantify brain infarction in rats exposed throughout gestation and lactation to the commercial PCB mixture Aroclor 1254 (A1254). Although our findings are paradoxical in that developmental PCB exposure was observed to protect against stroke damage, they are of public health significance in that they identify developmental exposures to environmental contaminants as potentially significant determinants of adult susceptibility to cerebral ischemia.

## Materials and Methods

### Animals and PCB exposures

All animals used in these studies were treated humanely and with regard for alleviation of suffering, and all protocols involving animals were approved by the Oregon Health & Science University Institutional Animal Care and Use Committee prior to the initiation of experimentation. Adult male and female Wistar rats were purchased from Charles River Laboratories (Hollister, CA) and housed individually, except during breeding, in standard plastic cages with Alpha-Dri bedding (Shepherd Specialty Papers, Watertown, TN) in a temperature-controlled room (22 ± 2°C) on a 12-hr reverse light–dark cycle. Food and water were provided *ad libitum*.

Dams used in the study delivered a litter of 10–15 pups (*n* = 5–7 dams per treatment group). On postnatal day (PND) 2, litters were culled to 10 pups. Pups were weaned on PND21. Dams were dosed with A1254 (lot 124-191; purity > 99%; AccuStandard, New Haven, CT) at 0.1 or 1 mg/kg/day beginning 2 weeks prior to breeding and continuing until PND21. A1254 diluted in HPLC-grade methanol was pipetted onto Keebler Golden Vanilla Wafers (Kellogg Company, Battle Creek, MI), which were fed to dams daily throughout the exposure period. Controls received wafers dosed with an equal volume of vehicle. Dams were observed to ensure the entire wafer was consumed (typically within 5 min), and pups were not allowed access to the wafers.

### Experimental stroke in rat

**Figure 1:**
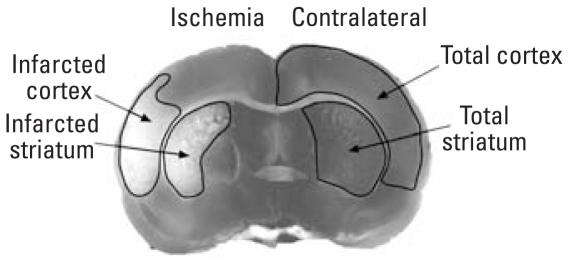
Method for identifying infarct area illustrated in a representative digital image of a TTC-stained coronal slice. Tissue infarction appears as the absence of TTC staining (left), and the contra-lateral cortex and striatum (right) were identified using defined anatomical markers (Paxinos and Watson 1986).

When offspring reached 225–275 g body weight (6–8 weeks for males; 8–10 weeks for females), we induced transient focal cerebral ischemia by middle cerebral artery occlusion (MCAO) using the intraluminal filament technique with halothane anesthesia in O_2_-enriched air, as previously described (Rusa et al. 1999). Tissue perfusion over the ipsilateral parietal cortex was monitored using a laser-Doppler probe (Moor Instruments Ltd., Oxford, UK) placed 6 mm lateral and 2 mm posterior to bregma. Vascular occlusion was maintained for 2 hr, then the occluding filament was withdrawn and the brain reperfused for 22 hr. This typically results in a core infarct limited to the ipsilateral cortex and striatum. After 22 hr of reperfusion, brains were collected, immediately sectioned (seven 2-mm-thick coronal sections per brain) and stained with 2,3,5-triphenyltetrazolium chloride (TTC), which is a commonly used and highly reproducible method for determining infarct volume in the brain (Dettmers et al. 1994; Hatfield et al. 1991; Okuno et al. 2001; Tureyen et al. 2004). In viable tissue, the TTC salt accepts a proton from succinate dehydrogenase in the inner mitochondrial membrane, which leaves the succinate dehydrogenase in a reduced state. Reduced succinate dehydrogenase forms a red insoluble product, formazan, causing brain tissue to develop a red hue. In contrast, infarcted areas of brain tissue cannot reduce succinate dehydrogenase and therefore remain pale. After TTC staining, the rostral and caudal sides of each coronal section were digitally photographed (Figure 1) and the infarct area quantified using MCID image analysis software (InterFocus Imaging Ltd., Cambridge, UK). We integrated rostral and caudal infarct areas across all seven sections from each brain to calculate the infarct volume. To normalize for poststroke edema, infarct volume in the hemisphere, cortex, and striatum is expressed as a percentage of the corresponding contralateral structures, which were identified using defined anatomical markers (Paxinos and Watson 1986).

### Hepatic cytochrome P450 activity

We analyzed 7-ethyoxyresorufin *O*-deethylase (EROD) and 7-pentoxyresorufin *O*-depentylase (PROD) activities in hepatic microsomes isolated from rats used in MCAO studies according to the method of Lubet (1990), as modified by Kennedy and Jones (1994) and Kono et al. (1999). We measured resorufin formation using a Fusion Universal Microplate Analyzer (Packard Instruments, Meriden, CT) with an excitation filter of 544 nm and an emission filter of 590 nm. Enzyme activities were normalized to protein concentration as determined using the BCA Protein Assay (Pierce, Rockford, IL).

### Congener-specific analysis of PCBs in brain tissue

Whole brains harvested from PND62 rats were stored at −80°C and thawed immediately before extraction, cleanup, and fractionation using gel permeation chromatography as previously described (Sethajintanin et al. 2004). We used tetrachloro-*m*-xylene (TCMX) in hexanes in all samples as the internal surrogate standard. Additional fortification samples spiked with a 20-ng/g PCB mixture were included in all batches. Specific PCB congeners were determined using gas chromatography with microelectron capture detection (Agilent 6890N Network GC system; Agilent Technologies, Santa Clara, CA) as previously described (Sethajintanin and Anderson 2006). To minimize batch-to-batch bias, samples from any designated group were typically analyzed in three different batches. Reagent and extraction blanks were included in every batch. Quality control samples represented 30–40% of a sample set and were processed and analyzed exactly the same as samples. The method detection limits were determined as 3 times the heights of coincident peaks observed for each compound in the control rat tissue. None of the target analytes were identified in the reagent or extraction blanks. Recovery of the internal surrogate TCMX ranged from 45 to 86%. The average percent recovery of fortified samples was 59.9–97.2% for target PCB congeners.

### Quantitative reverse transcriptase-polymerase chain reaction (qRT-PCR)

PND7 and PND50 rats were euthanized and the brains excised; cortices and caudate putamen were rapidly dissected, snap frozen, and stored at −80°C. Total RNA was isolated using RNeasy (QIAGEN, Valencia, CA); RNA amount and quality were determined by spectrophotometry and gel electrophoresis, respectively; and, cDNA was reverse transcribed from 500 ng total RNA (High Capacity cDNA Archive Kit; Applied Biosystems, Foster City, CA). Primers and probes specific for rat B-cell leukemia/lymphoma 2 (*Bcl2*, GenBank accession no. NM 016993; GenBank 2007) and 18S RNA were as previously described (Cheng et al. 2007). Primers and probes specific for rat cytochrome P450 2C11 (*Cyp2C11*, GenBank accession no. X79081) were designed using Primer Express (Applied Biosystems) and purchased from Integrated DNA Technologies (Coralville, IA): forward and reverse primer sequences were 5′-CGCCGTTTCTCCAT-CATGA-3′ and 5′-TCTTGAATACGGTC-CTCAATGGT-3′, respectively; probe sequence was 5′-/56-FAM/CTCTTGCC-CATCCCAAAAGTCCTCAGG/36 -TAMsp/-3′. We determined the amount of target gene in experimental samples by linear regression analyses using a standard curve generated for each target gene. Expression levels of target genes were normalized against endogenous 18S RNA levels.

### Plasma levels of sex steroids

After 22 hr of reperfusion, each animal was deeply anesthetized with halothane, and blood samples were obtained by cardiac puncture for measurement of plasma 17β-estradiol and testosterone by radioimmunoassay as previously described by Alkayed et al. (2000).

### Statistical analyses

We analyzed infarct volume by two-way analysis of variance (ANOVA) for factors of region and dose in each sex, and all other data were analyzed by ANOVA for treatment effects. If significant effects were identified (*p* < 0.05), post hoc analyses were performed using the Newman-Keuls multiple comparison test.

## Results

### Effects of A1254 treatment on maternal and fetal toxicity

**Figure 2:**
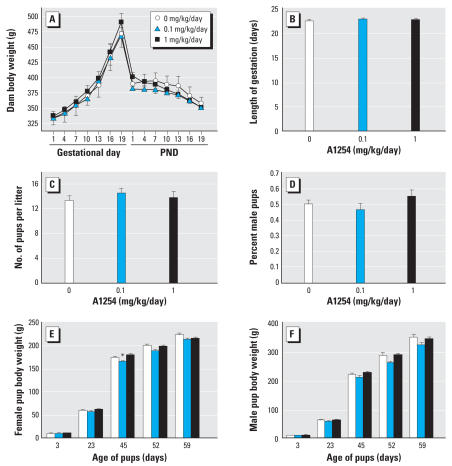
Effects of A1254 treatments [0 (vehicle; *n* = 7), 0.1 (*n* = 7), or 1 mg/kg (*n* = 6) on maternal or fetal toxicity. (*A*) Maternal body weight throughout gestation and lactation, (*B*) length of gestation, (*C*) litter size, (*D*) sex ratio, (*E*) body weight of female offspring (*n* ≥ 11 per treatment group), and (*F*) body weight of male offspring (*n* ≥ 11 per treatment group). See “Materials and Methods” for details. Data are presented as mean ± SE. *p < 0.05.

Exposure of dams to A1254 at 0.1 or 1.0 mg/kg/day did not alter maternal weight gain during gestation, maternal body weight during lactation, length of gestation, litter size, or sex ratio relative to vehicle controls (Figure 2A–D). Body weight of the offspring was similarly unaffected by developmental PCB exposure, with the exception of a transient decrease at PND45 in females in the 0.1-mg/kg/day treatment group (Figure 2E,F).

### Effect of developmental PCB exposure on infarct volume after ischemic stroke

**Figure 3:**
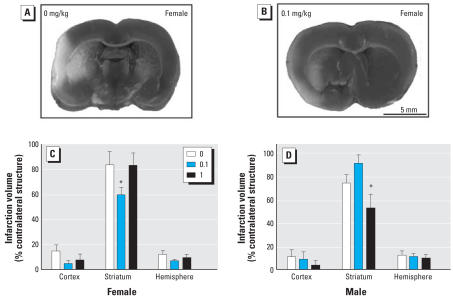
Effects of A1254 exposure [0 (vehicle), 0.1, or 1 mg/kg] on infarct volume measured after 22 hr of reperfusion following MCAO in female (8–10 weeks of age) and male rats (6–8 weeks of age); see “Materials and Methods” for details. Representative photomicrographs of TTC-stained coronal slices demonstrating infarcts (nonstained region) in the ipsilateral (left) cortex and striatum of females from vehicle control (*A*) and 0.1-mg/kg/day (*B*) treatment groups. Infarct size was significantly reduced in the striatum of females (*C*) and males (*D*) in the 0.1- and 1.0-mg/kg A1254 groups, respectively. Data are presented as the mean ± SE. *n* = 7–9 per treatment group. *p < 0.05.

To test the hypothesis that developmental PCB exposure influences adult sensitivity to ischemic brain injury, we performed transient MCAO in adult rats exposed throughout gestation and lactation to A1254 in the maternal diet. The survival rate was 100% in all treatment groups. Arterial blood pressure and gases, body temperature, and laser Doppler perfusion values were monitored throughout surgery, MCAO, and early reperfusion. Physiologic values obtained from the midpoint of MCAO were generally comparable between treatment groups (Table 1). While mean arterial blood pressure and blood glucose were significantly decreased in a subset of A1254 dose groups, these lower values were still well within the normal physiologic range for these parameters. However, relative to vehicle controls (Figure 3A), infarct volume was significantly decreased in both females and males exposed developmentally to A1254, although in females this effect was observed only in the lower A1254 dose group (Figure 3B,C) and in males only in the higher A1254 dose group (Figure 3D). Although similar trends were noted in the cortex and total hemisphere, significant treatment-related effects were limited to the striatum.

### Effects of developmental A1254 exposure on hepatic CYP activity and adult brain PCB levels

**Figure 4:**
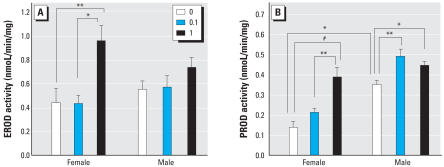
Effects of A1254 exposure [0 (vehicle), 0.1, or 1 mg/kg] on CYP activity in the liver of adult rats; see “Materials and Methods” for details. Activities of EROD (*A*) and PROD (*B*), biomarkers of exposure to coplanar and noncoplanar PCBs, respectively, were measured in hepatic microsomes from adult female (8–10 weeks of age) and male rats (6–8 weeks of age). Developmental PCB exposure caused a sex- and dose-dependent increase in both EROD and PROD activity. Data are presented as mean ± SE; *n* = 8 per treatment group per sex. *p < 0.05. **p < 0.01. ^#^*p* < 0.001.

A question raised by the stroke studies was why females were more sensitive to the neuroprotective effects of developmental A1254 exposure than males. One possible mechanism is differences in PCB metabolism between the sexes. As an indirect test of this hypothesis, we measured CYP activities in hepatic microsomes from animals receiving MCAO and quantified PCB brain levels in comparably aged litter-mates. In vehicle controls, EROD activity was similar between females and males; however, developmental A1254 exposure significantly increased EROD activity among females in the high-dose group but not among males in either A1254 dose group (Figure 4A). PROD activity was significantly higher among vehicle control males than among females, and developmental exposure to A1254 increased PROD activity in both females and males (Figure 4B). In females, this effect was observed only in the high-dose group, whereas in males, this effect was observed in both A1254 dose groups.

Of the 32 congeners chosen for analysis based on their toxicity, presence in A1254, abundance in environmental samples, and analytical capability, 31 were below the detection limit in brains of vehicle controls (Table 2); the one congener that was detected (PCB-153) was observed in only one of four samples, and the concentration in this sample was not significantly different from background. Interestingly, none of the 32 congeners were detected at levels significantly different from vehicle controls in brain tissue from either males or females exposed developmentally to A1254 at 0.1 mg/kg/day. In contrast, 12 congeners were detected at levels significantly increased relative to vehicle controls in brains of both males and females exposed developmentally to A1254 at 1 mg/kg/day. Detected congeners were predominantly *ortho*-substituted, and no significant differences were noted between sexes.

### *Effect of developmental A1254 exposure on* Bcl2 *and* Cyp2C11 *expression in the brain.*

**Figure 5:**
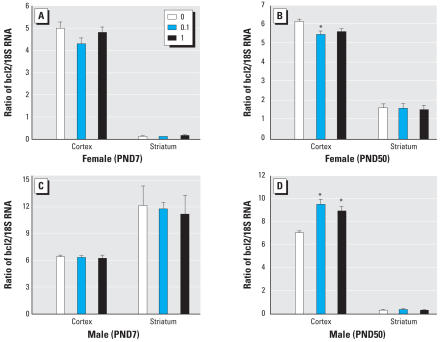
Changes in levels of transcript encoding *Bcl2* in rats after developmental exposure to A1254 (mg/kg/day). Total RNA was extracted from the cortex and striatum of females (*A*, *B*) and males (*C*, *D*) at PND7 (*A*, *C*) and PND50 (*B*, *D*). Bcl2 mRNA was quantified by qRT-PCR and normalized against endogenous 18S RNA. Developmental A1254 exposure caused age-, dose- and sex-specific changes in cortical expression of Bcl2 mRNA independent of PCB-related changes in infarct volume. Data are presented as mean ± SE; *n* = 5–6 per treatment group per sex. *p < 0.05.

**Figure 6:**
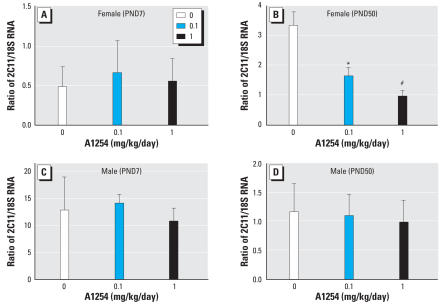
Correlation of PCB effects on Cyp2C11 mRNA levels and PCB effects on stroke outcome. Total RNA was extracted from the striatum of females (*A*, *B*) and males (*C*, *D*) at PND7 (*A*, *C*) and PND50 (*B*, *D*). Cyp2C11 mRNA was quantified by qRT-PCR and normalized against endogenous 18S RNA. Developmental exposure to A1254 at 0.1 or 1.0 mg/kg/day in the maternal diet significantly decreased Cyp2C11 mRNA expression in PND50 females (*B*), but had no effect on *Cyp2C11* transcripts in the striatum of PND7 females (*A*) or males of either age (*C*, *D*). Data are presented as mean ± SE; *n* = 5–6 per treatment group per sex. *p < 0.05. ^#^*p* < 0.001.

Developmental A1254 exposure had no effect on cortical or striatal levels of Bcl2 mRNA in either sex at PND7 (Figure 5A,C). In the PND50 brain, A1254 at 0.1 mg/kg/day reduced *Bcl2* expression in the female cortex (Figure 5B), whereas both A1254 doses increased *Bcl2* in the male cortex (Figure 5D). We next determined whether developmental A1254 exposure increased Cyp2C11 mRNA levels in the striatum of males or females at PND7 or PND50. The only significant effect was decreased *Cyp2C11* transcription in PND50 females exposed developmentally to either A1254 dose (Figure 6).

### Effect of developmental A1254 exposure on sex hormone levels during MCAO

To test the hypothesis that the neuroprotective effects of developmental A1254 exposure against cerebral ischemia are mediated by PCB effects on estrogen levels, we quantified plasma levels of estradiol and testosterone 22 hr after MCAO. The only treatment-related effect was a significant increase in plasma estradiol in females exposed developmentally to A1254 at 1 mg/kg/day; however, even these values were low and physiologically unremarkable.

## Discussion

In the present study, our data identify developmental exposure to environmental contaminants as a novel determinant of adult susceptibility to cerebral ischemia. Specifically, we demonstrate that developmental exposure to PCBs alters stroke outcome in adults, although in an unexpected manner, given previous epidemiologic and laboratory evidence suggesting that PCBs increase the incidence of risk factors for stroke, such as diabetes (Everett et al. 2007; Longnecker and Daniels 2001; Vasiliu et al. 2006) and vascular disease (Carpenter 2006; Hennig et al. 2005). Paradoxically, we observed that developmental PCB exposure provided protection against ischemic injury, evident as decreased infarct volume in adult male and female rats exposed to A1254 at 1.0 or 0.1 mg/kg/day in the maternal diet, respectively. Although these PCB exposure levels are higher than current background levels, they are about 3-fold lower than those reported in children exposed prenatally to PCB-contaminated seafood (Grandjean et al. 2001). Our congener-specific analyses of brains from adult rats exposed developmentally to A1254 at 1 mg/kg/day in the maternal diet identified 12 predominantly *ortho*-substituted congeners at concentrations ranging from 0.4 to 8.6 ng/g wet weight. Analyses of PCB levels in human brains obtained from the general adult population similarly identified predominantly *ortho*-substituted congeners at concentrations ranging from 0.07 to 8.0 ng/g wet weight (Chu et al. 2003; Covaci et al. 2002; Dewailly et al. 1999). Interestingly, our analyses did not detect PCB congeners above control levels in the brains of adult animals exposed developmentally to A1254 at 0.1 mg/kg/day, even though this dose was neuroprotective against focal cerebral ischemia in females, suggesting that PCB effects on stroke outcome may result from PCB interactions with developmental processes.

The mechanism(s) mediating the neuro-protective effect of PCBs appear to be independent of reported PCB effects on vascular function. PCBs have been shown to interfere with vascular function and induce microvessel formation outside the brain (Eum et al. 2006; Hennig et al. 2005; Tavolari et al. 2006), but laser-Doppler flowmetry (LDF) indicated that cerebral blood flow during MCAO was similar between treatment groups. Although mean arterial pressure (MAP) was within normal physiologic parameters across all treatments, we did observe a statistically significant decrease in MAP among females in both the low- and high-dose A1254 groups and among males in the high-dose A1254 group. However, it is unlikely that this contributes to the neuroprotective effects of PCBs because decreased MAP would be expected to worsen cerebral blood flow during MCAO, resulting in larger infarcts. We also observed a statistically significant decrease in plasma glucose among females in the 0.1-mg/kg/day A1254 dose group. Although hypoglycemia has been reported to induce ischemic tolerance in the brain (Bergstedt et al. 1993), this effect was observed only subsequent to severe insulin-induced hypoglycemia. Because blood glucose levels among females in the 0.1-mg/kg/day A1254 group were still within normal physiologic range, it seems unlikely that this is the mechanism by which PCBs confer protection against focal cerebral ischemia. Thus, the protective effects of PCBs in ischemic brain injury appear to be independent of PCB effects on the vasculature and homeostatic regulation of blood glucose, suggesting that although PCB exposure may increase risk factors for stroke incidence, such effects may be offset by PCB-induced neuroprotection that decreases the extent of damage caused by ischemic stroke.

Stroke outcome can be significantly altered by preconditioning (Koerner and Alkayed 2007) induced by either mild cerebral ischemic stress or exposure to noxious agents, including the endotoxin lipopolysaccharide (Deplanque and Bordet 2000), the oxidative phosphorylation inhibitor 3-nitropropionic acid (Nakase et al. 2000; Sugino et al. 1999; Wiegand et al. 1999), and ethanol (Wang et al. 2007). Mechanism(s) implicated in at least some types of preconditioning include increased *Bcl2* and *Cyp2C11* expression (Koerner and Alkayed 2007). We observed increased *Bcl2* expression in males at the protective developmental A1254 exposure (1.0 mg/kg/day), but it was also increased at the nonprotective developmental A1254 exposure (0.1 mg/kg/day), suggesting that *Bcl2* up-regulation alone does not mediate the neuroprotective effects of developmental PCB exposure. Moreover, *Bcl2* expression was reduced in females exposed to A1254 at the neuroprotective dose of 0.1 mg/kg/day, suggesting that *Bcl2* up-regulation is not required for PCB effects on infarct volume in these animals. Similarly, up-regulation of *Cyp2C11* expression in the brain is unlikely to mediate the neuroprotective effects of developmental A1254 exposure since PCB-related effects on *Cyp2C11* expression did not correlate with PCB-related decreases in infarct volume. However, these data do not rule out the possibility that PCBs confer protection against ischemic injury by preconditioning, because preconditioning can be elicited by mechanisms other than *Bcl2* and *Cyp2C11* up-regulation, including increased oxygen free radicals, calcium influx via *N*-methyl-D-aspartate (NMDA) receptors, and proinflammatory cytokines (Koerner and Alkayed 2007). How these signals confer protection is not completely understood, but working hypotheses include up-regulation of endogenous antioxidant mechanisms in the brain (Toyoda et al. 1997), such as superoxide dismutase (Bordet et al. 2000; Kato et al. 1995; Wada et al. 2001), and activation of transcription factors implicated in neuroprotection, such as cyclic AMP-response element binding protein (CREB) (Hara et al. 2003; Tauskela and Morley 2004) and nuclear factor-kappa B (NF-κB) (Pradillo et al. 2005; Ravati et al. 2001). Interestingly, PCBs have been shown to increase oxygen free radicals in cultured neurons (Mariussen and Fonnum 2006), enhance NMDA-mediated calcium influx (Gafni et al. 2004), and increase levels of proinflammatory cytokines in the brain (Goodwill et al. 2007). Whether these alternate mechanisms contribute to the neuro-protective actions of developmental PCB exposure against ischemic injury in the adult brain remains to be determined.

Alternatively, or in addition, PCB effects on infarct volume may be mediated by estrogenic mechanisms. Estrogen protects both male and female brains against ischemic damage (McCullough et al. 2001; Rusa et al. 1999). Although we did not observe A1254-induced changes in plasma concentrations of either estradiol or testosterone in animals undergoing MCAO, we cannot rule out the possibility that A1254 altered estrogen signaling via effects at the level of the estrogen receptor. PCBs compete with estrogen for binding to the estrogen receptor (Arcaro et al. 1999; DeCastro et al. 2006; Nesaretnam et al. 1996), and developmental PCB exposure has recently been reported to increase estrogen sensitivity (Ceccatelli et al. 2006) and to modulate sexual differentiation of the rodent brain (Colciago et al. 2006; Weiss 2002) When considered in the context of our observation that neuroprotection was conferred in the absence of detectable PCB levels in the female brain at the time of MCAO and emerging evidence that sex hormone effects on the developing brain may be important in establishing differences in stroke outcome between the sexes (Hurn et al. 2005), these observations suggest the intriguing hypothesis that developmental PCB exposure influences stroke outcome via modulation of the sexual differentiation of the developing brain.

Whether modulation of estrogenic signaling mediates the neuroprotective effects of developmental PCB exposure remains to be determined; however, such a mechanism suggests an explanation for the different dose–response relationship we observed between males and females. If the estrogenic properties of A1254 are additive with the protective actions of endogenous estrogen, this might explain why females are more sensitive to the protective effects of developmental A1254 exposure. The protection conferred in females was, however, lost at the higher developmental A1254 exposure. It has previously been reported that higher doses of estrogen are not effective in reducing injury (Noppens et al. 2005; Rusa et al. 1999; Wilson et al. 2000). Thus, exposure to A1254 at the higher dose possibly increases estrogen activity in females to an ineffective level, perhaps due to feedback inhibition. Consistent with this possibility, in males, which have lower levels of endogenous estrogen, A1254 conferred protection only at the higher exposure level. This seems a more plausible mechanism than the one we considered experimentally, which was a difference in PCB metabolism between sexes. Analyses of CYP activity in liver microsomes revealed that males in the vehicle control and 0.1-mg/kg/day A1254 exposure groups had increased PROD activity relative to females in comparable exposure groups. Therefore, males may metabolize noncoplanar PCBs more quickly than females, suggesting that a higher dose of A1254 may be required for an effect. However, congener-specific analyses of PCBs in adult brains did not reveal differences between sexes. Although it is possible that we may have missed critical differences between the sexes in the developing brain or in specific congeners not included in our analytical profile, these data suggest that differential PCB metabolism is not the principal mechanism contributing to sex differences in the dose–response relationship for developmental A1254 exposure effects on stroke outcome.

## Conclusions

Our data demonstrate that developmental A1254 exposure alters stroke outcome in the adult brain. Although the mechanisms mediating this effect remain unclear, these findings are important in that they establish developmental exposures to environmental contaminants as potentially significant determinants of the adult brain’s response to ischemic injury. These studies also identify developmental PCB exposure as a unique experimental tool for identifying novel therapeutic targets for limiting neuronal damage following ischemic injury. Determining which type(s) of PCBs contribute to the observed effects will improve assessments of PCB risks to human health, and identifying cellular and molecular mechanisms of PCB-induced neuroprotection will be necessary for translating these novel findings into potential stroke therapies.

## Figures and Tables

**Table 1:** Physiologic variables during MCAO.

	Females, A1254 treatment (mg/kg/day)			Males, A1254 treatment (mg/kg/day)		
Variable	0	0.1	1	0	0.1	1
MAP (mm Hg)	98 ± 4	86 ± 2*	87 ± 2*	111 ± 3	109 ± 3	99 ± 3*
Arterial blood pH	7.43 ± 0.01	7.42 ± 0.01	7.41 ± 0.01	7.46 ± 0.01	7.42 ± 0.02	7.43 ± 0.02
PaCO_2_ (mm Hg)	42 ± 2	44 ± 1	44 ± 3	41 ± 1	41 ± 2	44 ± 2
PaO_2_ (mm Hg)	122 ± 4	131 ± 4	130 ± 8	122 ± 2	136 ± 6	119 ± 6
Rectal temperature (°C)	37.0 ± 0.1	37.1 ± 0.1	37.0 ± 0.1	37.0 ± 0.1	37.0 ± 0.1	37.0 ± 0.1
Head temperature (°C)	37.1 ± 0.1	36.9 ± 0.1	36.8 ± 0.1	37.2 ± 0.1	36.9 ± 0.1	36.9 ± 0.1
Blood glucose (g/dL)	128 ± 6	109 ± 5*	131 ± 7	124 ± 5	108 ± 3	130 ± 9
LDF	40 ± 2	39 ± 2	39 ± 2	41 ± 2	35 ± 2	37 ± 2

Abbreviations: LDF, laser Doppler flowmetry; MAP, mean arterial blood pressure; PaCO_2_, arterial CO_2_ tension; PaO_2_, arterial O_2_ tension.

* *p* < 0.05 compared with vehicle treatment (ANOVA).

**Table 2:** Concentration of PCB congeners (ng/g wet weight) detected in brains of PND62 rats exposed developmentally to A1254.

	A1254 concentration (mg/kg/day)		
PCB congener	0 (vehicle)	0.1	1
1 *ortho* substitution			
PCB-105	BDL	BDL	2.3 ± 0.4#
PCB-118	BDL	BDL	6.3 ± 1.1#
PCB-156	BDL	BDL	1.9 ± 0.2#
2 *ortho* substitutions			
PCB-99	BDL	BDL	7.8 ± 2.4**
PCB-128	BDL	BDL	1.8 ± 0.4#
PCB-138	BDL	4.1 ± 2.0	8.6 ± 1.4**
PCB-153	1.20^a^	BDL	6.5 ± 1.0#
PCB-158	BDL	BDL	1.8 ± 0.2#
PCB-170	BDL	BDL	0.9 ± 0.2#
PCB-180	BDL	BDL	1.4 ± 0.1#
3 *ortho* substitutions			
PCB-183	BDL	BDL	0.4 ± 0.1**
PCB-187	BDL	BDL	1.0 ± 0.1#

BDL, below detection limit. *n* = 4 per treatment group.

a Concentration detected in one of four samples; the other three samples were BDL. Other congeners that were tested and found to be BDL in all samples include coplanar PCBs 37, 77, 126, and 169; mono-*ortho*-substituted PCBs 8, 28, 60, 66, 70, 74, 114, and 189; di-*ortho*-substituted

PCBs 44, 49, 52, 82, 87, 101, and 166; and PCB179, which has four *ortho* substitutions.

** *p* < 0.01.

# *p* < 0.001 (ANOVA).

**Table 3:** Estrogen and testosterone levels in plasma 22 hr after MCAO.

A1254 (mg/kg/day)	No.	Estradiol (pg/mL)	Testosterone (ng/mL)
Male			
0	8	18 ± 1	2.14 ± 0.67
0.1	8	20 ± 2	2.91 ± 1.22
1	7	22 ± 3	2.97 ± 0.50
Female			
0	8	18 ± 1	0.47 ± 0.06
0.1	8	24 ± 3	0.61 ± 0.05
1	9	27 ± 3*	0.51 ± 0.04

* *p* < 0.05 (ANOVA).

## References

[b1-ehp0116-000474] Alkayed NJ, Murphy SJ, Traystman RJ, Hurn PD, Miller VM (2000). Neuroprotective effects of female gonadal steroids in reproductively senescent female rats. Stroke.

[b2-ehp0116-000474] Arcaro KF, Yi L, Seegal RF, Vakharia DD, Yang Y, Spink DC (1999). 2,2’,6,6’-Tetrachlorobiphenyl is estrogenic *in vitro* and *in vivo*. J Cell Biochem.

[b3-ehp0116-000474] Barlow BK, Cory-Slechta DA, Richfield EK, Thiruchelvam M (2007). The gestational environment and Parkinson’s disease: evidence for neurodevelopmental origins of a neurodegenerative disorder. Reprod Toxicol.

[b4-ehp0116-000474] Basha MR, Braddy NS, Zawia NH, Kodavanti PR (2006). Ontogenetic alterations in prototypical transcription factors in the rat cerebellum and hippocampus following perinatal exposure to a commercial PCB mixture. Neurotoxicology.

[b5-ehp0116-000474] Bergstedt K, Hu BR, Wieloch T (1993). Initiation of protein synthesis and heat-shock protein-72 expression in the rat brain following severe insulin-induced hypoglycemia. Acta Neuropathol.

[b6-ehp0116-000474] Bolin CM, Basha R, Cox D, Zawia NH, Maloney B, Lahiri DK (2006). Exposure to lead and the developmental origin of oxidative DNA damage in the aging brain. FASEB J.

[b7-ehp0116-000474] Bordet R, Deplanque D, Maboudou P, Puisieux F, Pu Q, Robin E (2000). Increase in endogenous brain superoxide dismutase as a potential mechanism of lipopolysaccharide-induced brain ischemic tolerance. J Cereb Blood Flow Metab.

[b8-ehp0116-000474] Carpenter DO (2006). Polychlorinated biphenyls (PCBs): routes of exposure and effects on human health. Rev Environ Health.

[b9-ehp0116-000474] Ceccatelli R, Faass O, Schlumpf M, Lichtensteiger W (2006). Gene expression and estrogen sensitivity in rat uterus after developmental exposure to the polybrominated diphenylether PBDE 99 and PCB. Toxicology.

[b10-ehp0116-000474] Chen YC, Guo YL, Hsu CC, Rogan WJ (1992). Cognitive development of Yu-Cheng (“oil disease”) children prenatally exposed to heat-degraded PCBs. JAMA.

[b11-ehp0116-000474] Cheng J, Alkayed NJ, Hurn PD (2007). Deleterious effects of dihydrotestosterone on cerebral ischemic injury. J Cereb Blood Flow Metab.

[b12-ehp0116-000474] Chu S, Covaci A, Schepens P (2003). Levels and chiral signatures of persistent organochlorine pollutants in human tissues from Belgium. Environ Res.

[b13-ehp0116-000474] Colciago A, Negri-Cesi P, Pravettoni A, Mornati O, Casati L, Celotti F (2006). Prenatal Aroclor 1254 exposure and brain sexual differentiation: effect on the expression of testosterone metabolizing enzymes and androgen receptors in the hypothalamus of male and female rats. Reprod Toxicol.

[b14-ehp0116-000474] Covaci A, de Boer J, Ryan JJ, Voorspoels S, Schepens P (2002). Distribution of organobrominated and organochlorinated contaminants in Belgian human adipose tissue. Environ Res.

[b15-ehp0116-000474] Craft TK, Zhang N, Glasper ER, Hurn PD, Devries AC (2006). Neonatal factors influence adult stroke outcome. Psychoneuroendocrinology.

[b16-ehp0116-000474] DeCaprio AP, Johnson GW, Tarbell AM, Carpenter DO, Chiarenzelli JR, Morse GS (2005). Polychlorinated biphenyl (PCB) exposure assessment by multivariate statistical analysis of serum congener profiles in an adult Native American population. Environ Res.

[b17-ehp0116-000474] DeCastro BR, Korrick SA, Spengler JD, Soto AM (2006). Estrogenic activity of polychlorinated biphenyls present in human tissue and the environment. Environ Sci Technol.

[b18-ehp0116-000474] Deplanque D, Bordet R (2000). Pharmacological preconditioning with lipopolysaccharide in the brain. Stroke.

[b19-ehp0116-000474] Dettmers C, Hartmann A, Rommel T, Kramer S, Pappata S, Young A (1994). Immersion and perfusion staining with 2,3,5-triphenyltetrazolium chloride (TTC) compared to mitochondrial enzymes 6 hours after MCA-occlusion in primates. Neurol Res.

[b20-ehp0116-000474] Dewailly É, Mulvad G, Pedersen HS, Ayotte P, Demers A, Weber JP (1999). Concentration of organochlorines in human brain, liver, and adipose tissue autopsy samples from Greenland. Environ Health Perspect.

[b21-ehp0116-000474] Eum SY, Rha GB, Hennig B, Toborek M (2006). c-Src is the primary signaling mediator of polychlorinated biphenyl-induced interleukin-8 expression in a human microvascular endothelial cell line. Toxicol Sci.

[b22-ehp0116-000474] Everett CJ, Frithsen IL, Diaz VA, Koopman RJ, Simpson WM, Mainous AG (2007). Association of a polychlorinated dibenzo-*p*-dioxin, a polychlorinated biphenyl, and DDT with diabetes in the 1999–2002 National Health and Nutrition Examination Survey. Environ Res.

[b23-ehp0116-000474] Gafni J, Wong PW, Pessah IN (2004). Non-coplanar 2,2’,3,5’,6-pentachlorobiphenyl (PCB 95) amplifies ionotropic glutamate receptor signaling in embryonic cerebellar granule neurons by a mechanism involving ryanodine receptors. Toxicol Sci.

[b24-ehp0116-000474] GenBank (2007). GenBank Overview.

[b25-ehp0116-000474] Goodwill MH, Lawrence DA, Seegal RF (2007). Polychlorinated biphenyls induce proinflammatory cytokine release and dopaminergic dysfunction: protection in interleukin-6 knockout mice. J Neuroimmunol.

[b26-ehp0116-000474] Grandjean P, Weihe P, Burse VW, Needham LL, Storr-Hansen E, Heinzow B (2001). Neurobehavioral deficits associated with PCB in 7-year-old children prenatally exposed to seafood neurotoxicants. Neurotoxicol Teratol.

[b27-ehp0116-000474] Hara T, Hamada J, Yano S, Morioka M, Kai Y, Ushio Y (2003). CREB is required for acquisition of ischemic tolerance in gerbil hippocampal CA1 region. J Neurochem.

[b28-ehp0116-000474] Hatfield RH, Mendelow AD, Perry RH, Alvarez LM, Modha P (1991). Triphenyltetrazolium chloride (TTC) as a marker for ischaemic changes in rat brain following permanent middle cerebral artery occlusion. Neuropath Appl Neurobiol.

[b29-ehp0116-000474] Hennig B, Reiterer G, Majkova Z, Oesterling E, Meerarani P, Toborek M (2005). Modification of environmental toxicity by nutrients: implications in atherosclerosis. Cardiovasc Toxicol.

[b30-ehp0116-000474] Hsu PC, Pan MH, Li LA, Chen CJ, Tsai SS, Guo YL (2007). Exposure in utero to 2,2’,3,3’,4,6’-hexachlorobiphenyl (PCB 132) impairs sperm function and alters testicular apoptosis-related gene expression in rat offspring. Toxicol Appl Pharmacol.

[b31-ehp0116-000474] Hurn PD, Vannucci SJ, Hagberg H (2005). Adult or perinatal brain injury: does sex matter?. Stroke.

[b32-ehp0116-000474] Jursa S, Chovancova J, Petrik J, Loksa J (2006). Dioxin-like and non-dioxin-like PCBs in human serum of Slovak population. Chemosphere.

[b33-ehp0116-000474] Kato H, Kogure K, Araki T, Liu XH, Kato K, Itoyama Y (1995). Immunohistochemical localization of superoxide dismutase in the hippocampus following ischemia in a gerbil model of ischemic tolerance. J Cereb Blood Flow Metab.

[b34-ehp0116-000474] Kennedy SW, Jones SP (1994). Simultaneous measurement of cytochrome P4501A catalytic activity and total protein concentration with a fluorescence plate reader. Anal Biochem.

[b35-ehp0116-000474] Kitagawa K (2007). CREB and cAMP response element-mediated gene expression in the ischemic brain. FEBS J.

[b36-ehp0116-000474] Koerner IP, Alkayed NJ, Bhardwaj A, Alkayed NJ, Kirsch JR, Traystman RJ (2007). Ischemic preconditioning. Acute Stroke: Bench to Bedside. Neurological Disease and Therapy Series.

[b37-ehp0116-000474] Kono H, Bradford BU, Yin M, Sulik KK, Koop DR, Peters JM (1999). CYP2E1 is not involved in early alcohol-induced liver injury. Am J Physiol.

[b38-ehp0116-000474] Kostyniak PJ, Hansen LG, Widholm JJ, Fitzpatrick RD, Olson JR, Helferich JL (2005). Formulation and characterization of an experimental PCB mixture designed to mimic human exposure from contaminated fish. Toxicol Sci.

[b39-ehp0116-000474] Kramer HJ, Drenth H, Fleuren R, Hengeveld S, Seinen W, VandenBerg M (1999). Metabolic rate constants of Ugilec 141 isomers and polychlorinated biphenyl congeners using rat hepatic microsomes and the identification of involved cytochrome P450 enzymes. Organohalogen Compounds.

[b40-ehp0116-000474] Longnecker MP, Daniels JL (2001). Environmental contaminants as etiologic factors for diabetes. Environ Health Perspect.

[b41-ehp0116-000474] Lubet RA, Guengerich FP, Nims RW (1990). The induction of alkoxyresorufin metabolism: a potential indicator of environmental contamination. Arch Environ Contamin Toxicol.

[b42-ehp0116-000474] Mariussen E, Fonnum F (2006). Neurochemical targets and behavioral effects of organohalogen compounds: an update. Crit Rev Toxicol.

[b43-ehp0116-000474] Mattson MP (2007). Calcium and neurodegeneration. Aging Cell.

[b44-ehp0116-000474] McCullough LD, Alkayed NJ, Traystman RJ, Williams MJ, Hurn PD (2001). Postischemic estrogen reduces hypo-perfusion and secondary ischemia after experimental stroke. Stroke.

[b45-ehp0116-000474] McMillen IC, Robinson JS (2005). Developmental origins of the metabolic syndrome: prediction, plasticity, and programming. Physiol Rev.

[b46-ehp0116-000474] Meerts IA, Hoving S, van den Berg JH, Weijers BM, Swarts HJ, van der Beek EM (2004). Effects of in utero exposure to 4-hydroxy-2,3,3’,4’,5-pentachlorobiphenyl (4-OH-CB107) on developmental landmarks, steroid hormone levels, and female estrous cyclicity in rats. Toxicol Sci.

[b47-ehp0116-000474] Morley R (2006). Fetal origins of adult disease. Sem Fetal Neonat Med.

[b48-ehp0116-000474] Nakase H, Heimann A, Uranishi R, Riepe MW, Kempski O (2000). Early-onset tolerance in rat global cerebral ischemia induced by a mitochondrial inhibitor. Neurosci Lett.

[b49-ehp0116-000474] Nesaretnam K, Corcoran D, Dils RR, Darbre P (1996). 3,4,3’,4’-Tetra-chlorobiphenyl acts as an estrogen in vitro and in vivo. Mol Endocrinol.

[b50-ehp0116-000474] Noppens RR, Kofler J, Hurn PD, Traystman RJ (2005). Dose-dependent neuroprotection by 17beta-estradiol after cardiac arrest and cardiopulmonary resuscitation. Crit Care Med.

[b51-ehp0116-000474] Okuno S, Nakase H, Sakaki T (2001). Comparative study of 2,3,5-triphenyltetrazolium chloride (TTC) and hematoxylin-eosin staining for quantification of early brain ischemic injury in cats. Neurol Res.

[b52-ehp0116-000474] Paxinos G, Watson C (1986). The Rat Brain in Stereotaxic Coordinates.

[b53-ehp0116-000474] Pradillo JM, Romera C, Hurtado O, Cardenas A, Moro MA, Leza JC (2005). TNFR1 upregulation mediates tolerance after brain ischemic preconditioning. J Cereb Blood Flow Metab.

[b54-ehp0116-000474] Ravati A, Ahlemeyer B, Becker A, Klumpp S, Krieglstein J (2001). Preconditioning-induced neuroprotection is mediated by reactive oxygen species and activation of the transcription factor nuclear factor-kappaB. J Neurochem.

[b55-ehp0116-000474] Rusa R, Alkayed NJ, Crain BJ, Traystman RJ, Kimes AS, London ED (1999). 17beta-estradiol reduces stroke injury in estrogen-deficient female animals. Stroke.

[b56-ehp0116-000474] Schantz SL, Widholm JJ, Rice DC (2003). Effects of PCB exposure on neuropsychological function in children. Environ Health Perspect.

[b57-ehp0116-000474] Sethajintanin D, Anderson KA (2006). Temporal bioavailability of organochlorine pesticides and PCBs. Environ Sci Technol.

[b58-ehp0116-000474] Sethajintanin D, Johnson ER, Loper BR, Anderson KA (2004). Bioaccumulation profiles of chemical contaminants in fish from the lower Willamette River, Portland Harbor, Oregon. Arch Envrion Contam Toxicol.

[b59-ehp0116-000474] Shcherbatykh I, Huang X, Lessner L, Carpenter DO (2005). Hazardous waste sites and stroke in New York State. Environ Health 4.

[b60-ehp0116-000474] Stewart P, Darvill T, Lonky E, Reihman J, Pagano J, Bush B (1999). Assessment of prenatal exposure to PCBs from maternal consumption of Great Lakes fish: an analysis of PCB pattern and concentration. Environ Res.

[b61-ehp0116-000474] Sugino T, Nozaki K, Takagi Y, Hashimoto N (1999). 3-Nitropropionic acid induces ischemic tolerance in gerbil hippocampus in vivo. Neurosci Lett.

[b62-ehp0116-000474] Tauskela JS, Morley P (2004). On the role of Ca^2+^ in cerebral ischemic preconditioning. Cell Calcium.

[b63-ehp0116-000474] Tavolari S, Bucci L, Tomasi V, Guarnieri T (2006). Selected poly-chlorobiphenyls congeners bind to estrogen receptor alpha in human umbilical vascular endothelial (HUVE) cells modulating angiogenesis. Toxicology.

[b64-ehp0116-000474] Toyoda T, Kassell NF, Lee KS (1997). Induction of ischemic tolerance and antioxidant activity by brief focal ischemia. Neuroreport.

[b65-ehp0116-000474] Tureyen K, Vemuganti R, Sailor KA, Dempsey RJ (2004). Infarct volume quantification in mouse focal cerebral ischemia: a comparison of triphenyltetrazolium chloride and cresyl violet staining techniques. J Neurosci Methods.

[b66-ehp0116-000474] Vasiliu O, Cameron L, Gardiner J, Deguire P, Karmaus W (2006). Polybrominated biphenyls, polychlorinated biphenyls, body weight, and incidence of adult-onset diabetes mellitus. Epidemiology.

[b67-ehp0116-000474] Wada K, Miyazawa T, Nomura N, Tsuzuki N, Nawashiro H, Shima K (2001). Preferential conditions for and possible mechanisms of induction of ischemic tolerance by repeated hyperbaric oxygenation in gerbil hippocampus. Neurosurgery.

[b68-ehp0116-000474] Wang Q, Sun AY, Simonyi A, Kalogeris TJ, Miller DK, Sun GY (2007). Ethanol preconditioning protects against ischemia/reperfusion-induced brain damage: role of NADPH oxidase-derived ROS. Free Radic Biol Med.

[b69-ehp0116-000474] Weiss B (2002). Sexually dimorphic nonreproductive behaviors as indicators of endocrine disruption. Environ Health Perspect.

[b70-ehp0116-000474] Wiegand F, Liao W, Busch C, Castell S, Knapp F, Lindauer U (1999). Respiratory chain inhibition induces tolerance to focal cerebral ischemia. J Cereb Blood Flow Metab.

[b71-ehp0116-000474] Wilson ME, Dubal DB, Wise PM (2000). Estradiol protects against injury-induced cell death in cortical explant cultures: a role for estrogen receptors. Brain Res.

[b72-ehp0116-000474] Zawia NH, Basha MR (2005). Environmental risk factors and the developmental basis for Alzheimer’s disease. Rev Neurosci.

